# Supplementary material for: A reappraisal of the role of the mammillothalamic tract in memory deficits following stroke in the thalamus
Source: Imaging Neurosci (Camb). 2025 Nov 12;3:IMAG.a.1007. doi: 10.1162/IMAG.a.1007 (PMC12612100; doi:10.1162/IMAG.a.1007)
Supplement: Supplementary Material [file IMAG.a.1007_supp.pdf]

A Reappraisal of the Role of the Mammillothalamic Tract in Memory Deficits Following Stroke in the Thalamus

Supplementary Figure

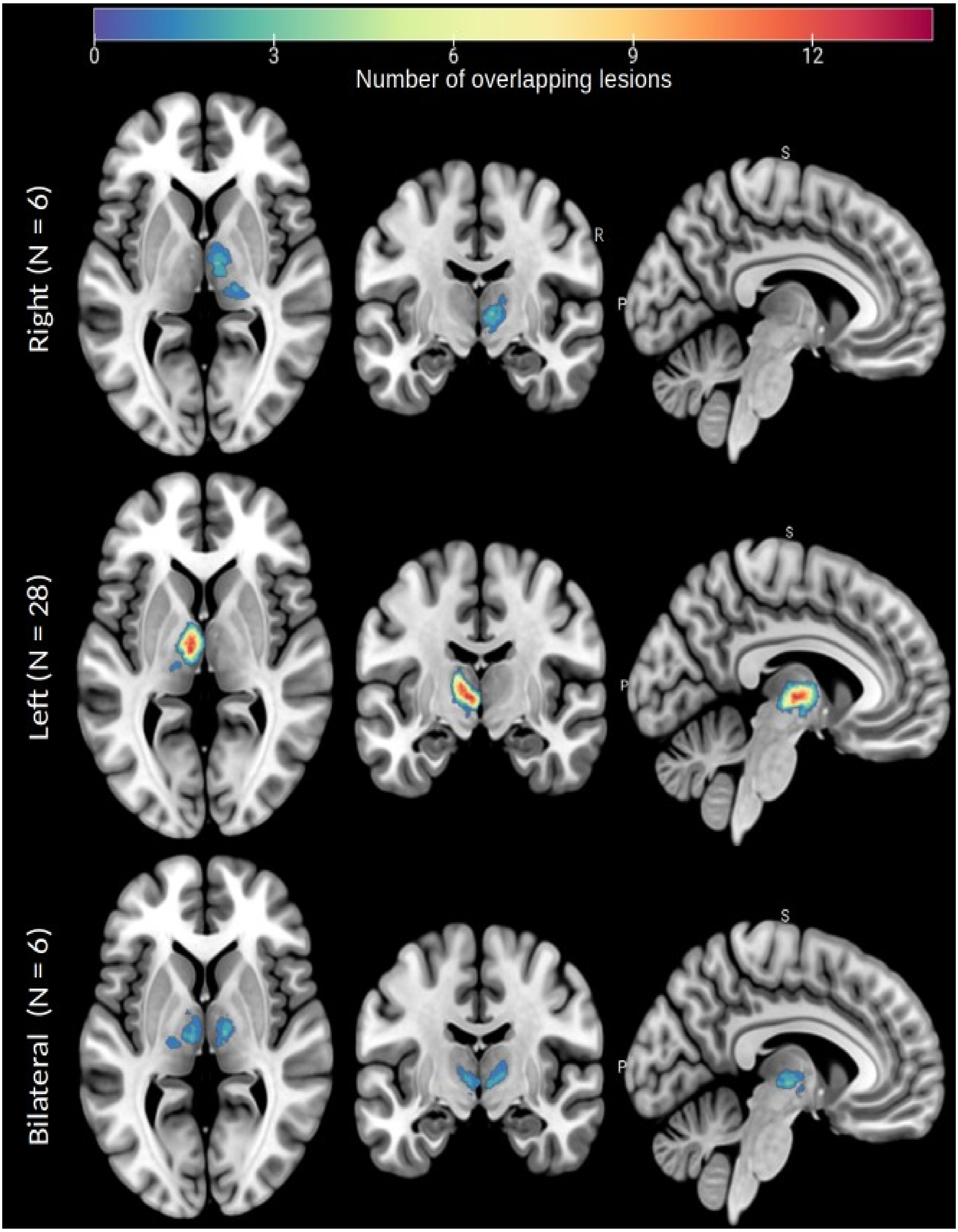

Supplementary figure 1: Representation of all lesions from the 40 patients on the MNI152 template after normalization and by infarct laterality. R: Right; S: Superior; P: Posterior

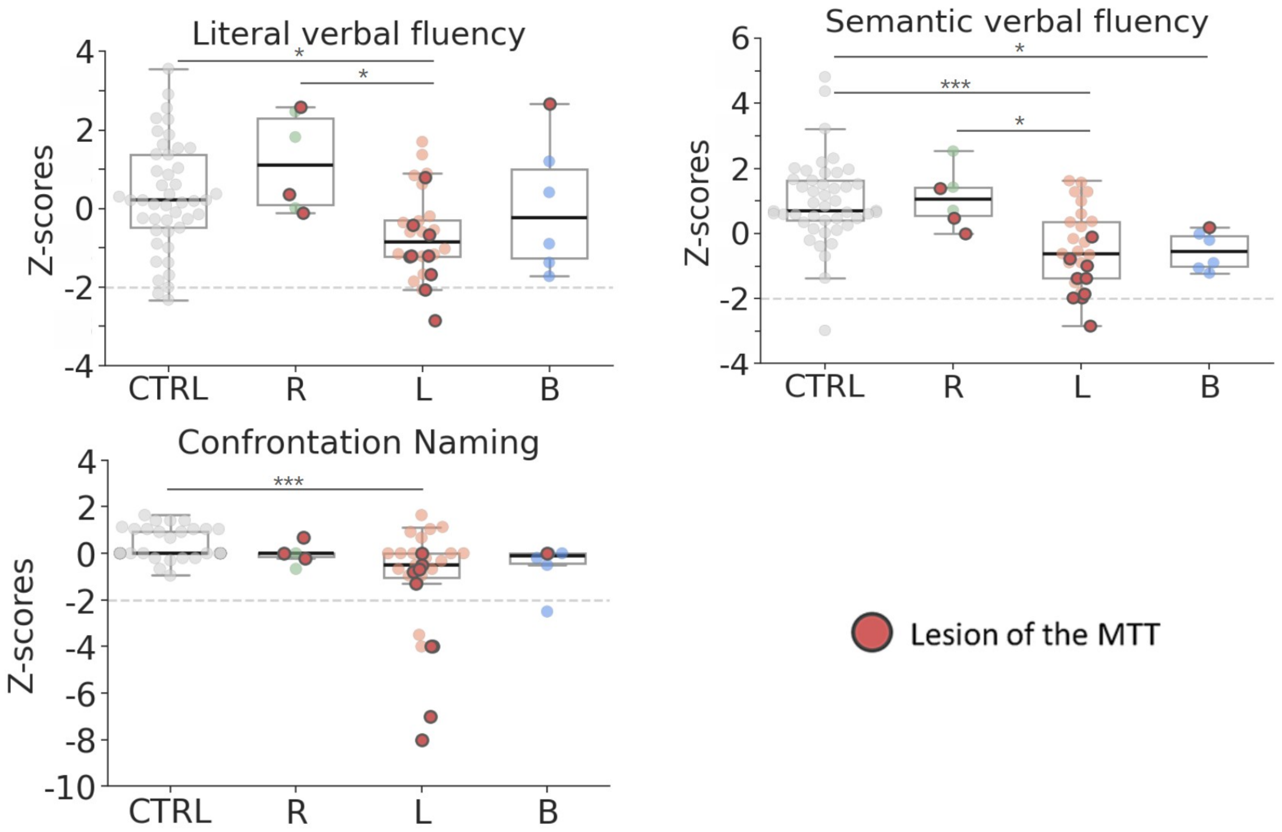

*Supplementary figure 2: Boxplots displaying Z-scores for literal and semantic verbal fluency, and for the confrontation naming test, by group and infarct laterality (healthy subjects = 45; Right = 6, Left = 28, Bilateral = 6). The black line within each box indicates the median. The gray dashed line highlights a z-score of -2 SD usually thresholding significant deficits. Red rounds represent patients with a lesion in the MTT. Statistical significance was assessed using Bonferroni-corrected Dunn's test: *p < 0.05, **p < 0.01, ***p < 0.001.*

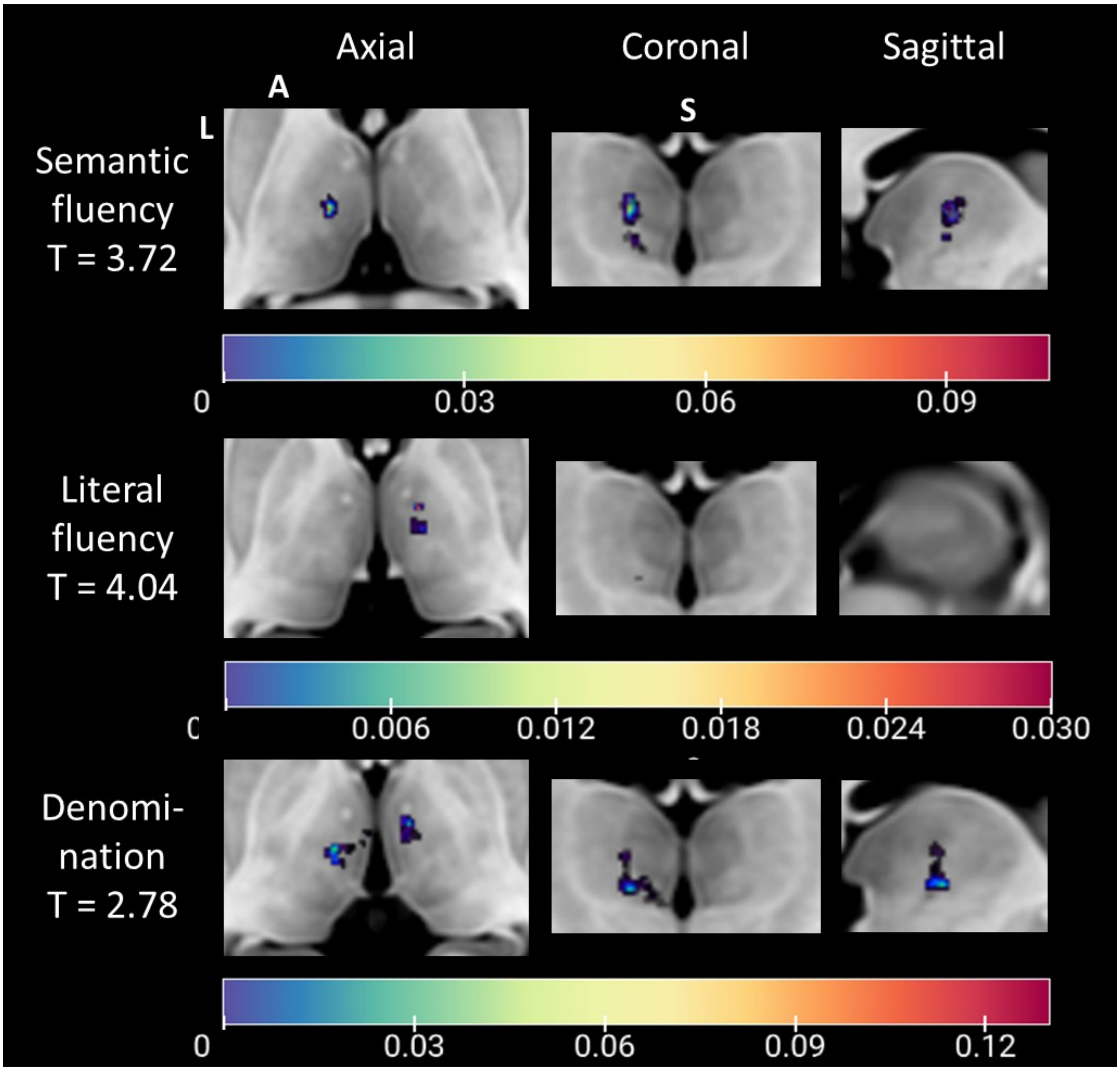

Supplementary figure 3: T-map of lesioned voxel clusters associated with deficits in Semantic Fluency, Literal Fluency or Confrontation Naming tests overlaid on an MNI152 slice. The T-value (T) represents the threshold for significance, which was not exceeded in any of these tests as reflected by the scales. L: Left, A: Anterior, S: Superior.

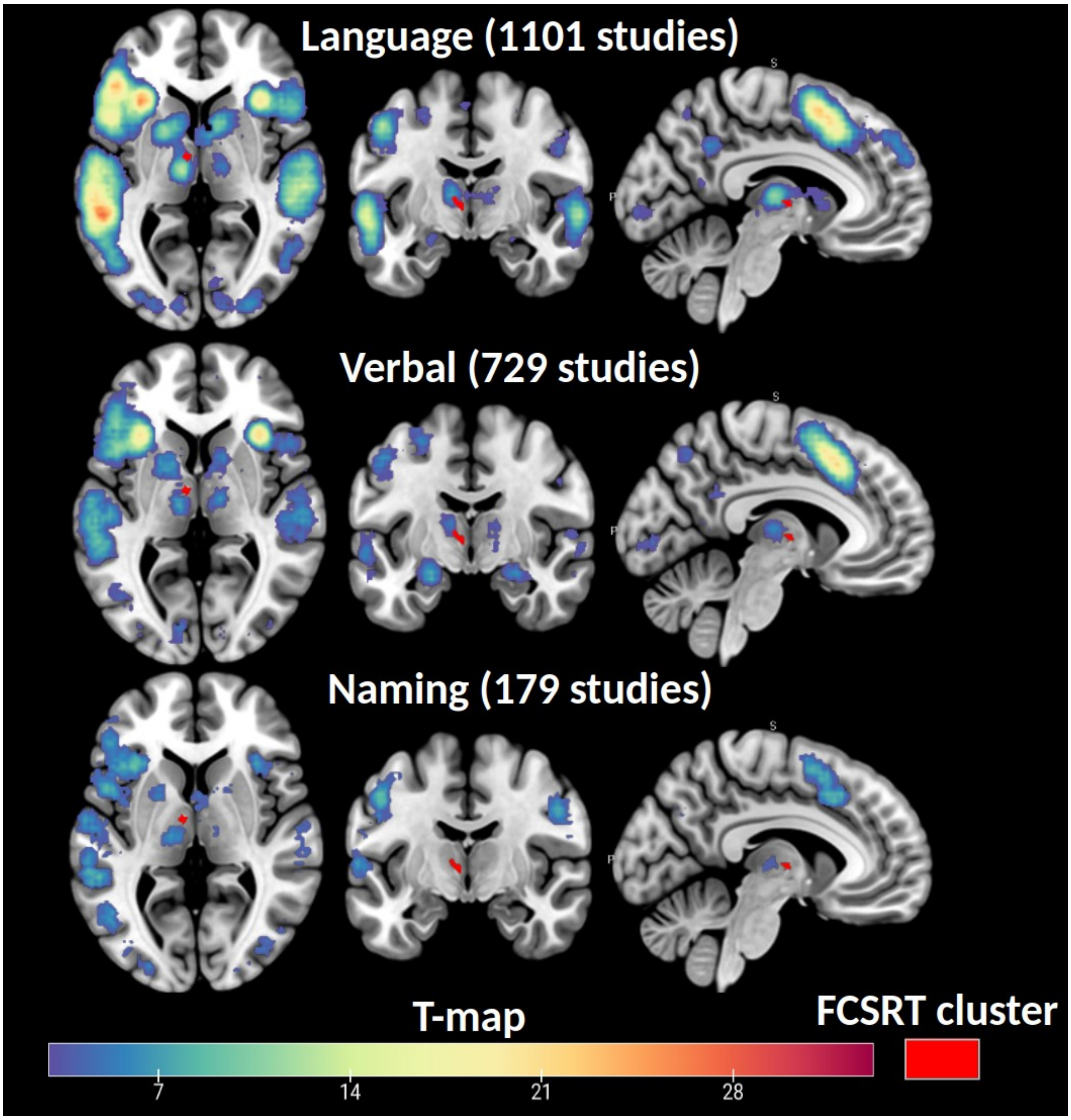

Supplementary figure 4: Visual representation of results from the Neurosynth query tool, displayed as T-maps of significant voxels associated with memory-related keywords from fMRI human studies. The cluster of voxels associated with poorer performance on the FCSRT Delayed Free Recall subtest is overlaid in red for comparison.
